# Light as a Broad-Spectrum Antimicrobial

**DOI:** 10.3389/fmicb.2018.00119

**Published:** 2018-02-02

**Authors:** Peter J. Gwynne, Maurice P. Gallagher

**Affiliations:** School of Biology, University of Edinburgh, Edinburgh, United Kingdom

**Keywords:** antimicrobials, infection, resistance, phototherapy, photosensitizers, blue light, ultraviolet, infrared

## Abstract

Antimicrobial resistance is a significant and growing concern. To continue to treat even simple infections, there is a pressing need for new alternative and complementary approaches to antimicrobial therapy. One possible addition to the current range of treatments is the use of narrow-wavelength light as an antimicrobial, which has been shown to eliminate a range of common pathogens. Much progress has already been made with blue light but the potential of other regions of the electromagnetic spectrum is largely unexplored. In order that the approach can be fully and most effectively realized, further research is also required into the effects of energy dose, the harmful and beneficial impacts of light on eukaryotic tissues, and the role of oxygen in eliciting microbial toxicity. These and other topics are discussed within this perspective.

## Introduction

The rise of antibiotic resistance has been (Jawetz, 1963; Lyon and Skurray, 1987; Neu, 1992) and continues to be (Goff et al., 2017; Manaia, 2017; Schroeder et al., 2017) extensively reported. Although new antibiotics are still being discovered (Ling et al., 2015; Zipperer et al., 2016), new discoveries are increasingly challenging and success in clinical trials is rare. In addition, the prevalence of pre-existing resistance systems in the environment (Bhullar et al., 2012), and the rapid rate of bacterial evolution (von Wintersdorff et al., 2016) mean that, even if adopted clinically, such compounds will only ever constitute a temporary reprieve. Thus, there is a clear need for alternative antimicrobial therapies which can be effective and sustainable in the longer term.

To prevent the emergence of resistance and to maximize treatment efficacy, novel therapies should ideally impact on a range of cellular targets. Whereas resistance to traditional antibiotics can arise through alteration of just a single amino acid residue in the antimicrobial target (Vila et al., 1994; Tsiodras et al., 2001), resistance becomes considerably less likely where a range of processes are targeted. The appeal of such a strategy is obvious and exemplified by broad-spectrum disinfectants (Russell, 2003).

**FIGURE 1:**
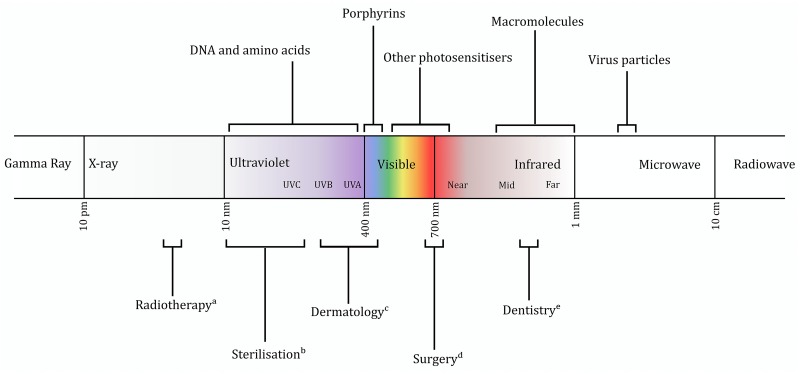
Representation of the electromagnetic spectrum, with regions of interest discussed in the text indicated. Current applications of certain wavebands also shown. (a) (Hill et al., 2014); (b) (Chang et al., 1985); (c) (Tanzi et al., 2003); (d) (Jin et al., 2010); (e) (Cobb, 2006).

Being widely found in a range of medical applications (**Figure 1**), electromagnetic radiation offers a promising avenue as an abiotic form of antimicrobial therapy. Currently two distinct light-mediated bactericidal techniques have been widely studied. The first of these, photodynamic therapy, has shown great potential against numerous pathogens and uses light of specific wavelength to stimulate an exogenously supplied photosensitizer, eliciting formation of toxic levels of reactive oxygen intermediates (Wainwright et al., 2016). In this perspective, we focus on the alternative approach in which light directly interacts with endogenous photosensitizers of the target microbe. This approach – eliminating the requirement for an additional third factor – removes a level of complexity in research, regulation, and application. It does, however, require a detailed knowledge of the interactions of biological systems (both prokaryotic and eukaryotic) with light.

## The Paradigm of Blue Light

The 1903 Nobel Prize was awarded to Niels Ryberg Finsen for the use of blue light (Møller et al., 2005) in the treatment of tuberculosis of the skin. Having been largely neglected through the subsequent era of antibiotic discovery, interest in antimicrobial light was renewed toward the end of the 20th century. Blue light [typically 400–450 nm (**Figure 1**)], which is absorbed by porphyrins and is thought to cause cell death by the generation of toxic reactive oxygen species, has largely remained the focus of research since the 1980s (Kjeldstad and Johnsson, 1986; Koenig et al., 1992). While initial experiments required addition of exogenous porphyrins (Bertoloni et al., 1984; Nitzan et al., 1987) or enhancement of endogenous porphyrin production (Sailer et al., 1997; van der Meulen et al., 1997), it latterly became clear that natural levels of porphyrin are sufficient to elicit toxicity (Ashkenazi et al., 2003).

The bactericidal effect of blue light has been shown in many pathogenic species (Gupta et al., 2015; Halstead et al., 2016). Energy doses in the 10 or 100s of J cm^-2^ are typically sufficient to kill *Staphylococcus aureus*, for example (Maclean et al., 2008a; Halstead et al., 2016). Moreover, while few studies have rigorously explored the kinetics of killing using blue light, there appears to be a correlation between energy dose and reduction in viability, suggesting that total energy (rather than power, duration, or wavelength) is the major factor (Maclean et al., 2008b; Ramakrishnan et al., 2014).

The oxygen-dependence of the antimicrobial effect has been demonstrated repeatedly (Gourmelon et al., 1994; Feuerstein et al., 2005). However, experiments in a high-oxygen environment showed no additional benefit (Bumah et al., 2015), suggesting that oxygen availability does not limit toxicity. The limiting factor seems likely to be the concentration of absorptive porphyrins: efficacy can be enhanced by induction of porphyrin production, and toxicity in different species has been found to correlate with their accumulation of the pigment (Nitzan et al., 2004; Hamblin et al., 2005; Choi et al., 2011). More recent studies have investigated the precise contribution of different porphyrin species as well as other photosensitizers such as flavins and nicotinamides (Cieplik et al., 2014; Battisti et al., 2017; Kim and Yuk, 2017).

Oxidative damage may not be the sole cause of cell death, however. It has long been suggested that other mechanisms may contribute (Kjeldstad, 1987; Henry et al., 1995), and oxygen scavengers cannot completely protect against toxicity (Feuerstein et al., 2005; Maclean et al., 2008b). In addition to damage to protein and lipid components, infrared spectroscopy has revealed that DNA cleavage caused by blue light is similar to that seen in UVA-treated cells (Bumah et al., 2016), which is unsurprising given the spectral proximity of UVA and blue light (**Figure 1**). A completely different mechanism of toxicity was suggested by a recent transcriptomic study, which implicated the upregulation of phage proteins after irradiation (Yang et al., 2017). Inhibition of phage maturation completely prevented cell death, suggesting that this pathway (or components thereof) may be of great significance. A phage-dependent mechanism has important implications for antimicrobial selectivity, although may also limit the possible spectrum of targets.

## Therapeutic Potential of Blue Light

While studies in the 1980s and 1990s typically focused on *Propionibacterium acnes*, recent research has been largely focused on *Staphylococcus aureus*. The immediate appeal of both organisms is their colonization of the skin, which is easily illuminated, although it is notable that one of the few published patient trials was carried out against *Helicobacter pylori* infection of the stomach (Lembo et al., 2009). While relatively little research has been translated into human clinical trials to date, animal models have been established (Yang et al., 2017; Zhu et al., 2017), demonstrating blue light killing of infecting cells a few hours after inoculation. These models are an encouraging development and further experiments showing successful treatment of an established infection featuring biofilm, persister cells, and intracellular bacteria will be a significant step toward clinical application.

Promisingly, however, reduction of cell numbers in established biofilms has been shown *in vitro* (Halstead et al., 2016; Wang et al., 2016). Tissue models also offer encouraging signs. Selectivity of the toxic effect for bacteria over mammalian cells has been demonstrated (Dai et al., 2013; Ramakrishnan et al., 2014). Different cell types appear variably tolerant to blue light, however: osteoblasts were killed above 36 J cm^-2^ whereas keratinocytes survived > 100 J cm^-2^. Given this difference in sensitivity across cell types, dose may have to be tailored depending on specific clinical application.

Currently few studies have investigated in detail the relationship between energy dose and killing. The available data (Maclean et al., 2008a, 2009; Endarko et al., 2012) suggest a sigmoidal dose–response curve implying that, as similarly observed with low-level oxidative stress (Kumar and Imlay, 2013), a sub-lethal light dose may be indefinitely tolerated by organisms with appropriate detoxifying systems. The existence of adaptive tolerance is supported by the finding that growth in low levels of blue light protects somewhat against subsequent high-intensity challenge (Tomb et al., 2017). With repeated sub-lethal dosage, resistance to blue light has been reported (Guffey et al., 2013) although this point remains contentious (de Sousa N.T. et al., 2015; Tomb et al., 2017). The importance of appropriate dosing and considerations of light transmission through tissue are clearly of particular importance given that blue light can promote biofilm formation (Tschowri et al., 2009; Mussi et al., 2010). Further work to understand the mechanisms of killing and the dose–response relationships is needed to provide a quantitative basis for widespread and effective implementation.

Although therapeutic treatment of established infections may be the primary aim, preventative intervention may also be of value and more readily achievable. Toward this aim, extended exposure at low (mW) power has been shown to slow bacterial growth (de Sousa D.L. et al., 2015; Ramakrishnan et al., 2016). The technology has also been trialed in a hospital setting, producing modest reductions in bacterial counts on surfaces (Maclean et al., 2013).

## Alternative Photosensitizers

Several factors may complicate the widespread use of blue light. Sensitivity varies across species (Maclean et al., 2009) and has been shown to be dependent on the accumulation of particular intracellular porphyrins (Hamblin et al., 2005). Indeed, even within the same species, susceptibility can vary (Kim and Yuk, 2017) and both porphyrin accumulation and subsequent toxicity are affected by growth medium (Henry et al., 1995). Blue light is also absorbed strongly by many mammalian cell types, limiting its tissue penetration (**Table 1**) and therefore application to surface tissues. Additionally, mammalian cells have been shown to produce reactive oxygen under blue light illumination (Ramakrishnan et al., 2016), and singlet oxygen is a known mutagen (Hiraku et al., 2007), suggesting that power levels will require careful titration to avoid damage to tissues. Longer wavelengths, by contrast, are more readily transmissible.

**Table 1:** Optical properties of selected wavelengths in skin.

Wavelength (nm)	Absorption coefficient in skin (cm^-1^)	Scattering coefficient in skin (cm^-1^)	Approx penetration depth (μm)	Major interactions in tissue
300	45.0	260.0	6.0	Mutagenic
350	25.0	220.0	60.0	Mutagenic
400	13.5	34.3	90.0	Mutagenic, photochemical
500	6.2	25.1	230.0	Photochemical
600	3.8	18.6	550.0	Photochemical
700	2.4	14.8	750.0	Photochemical, thermal
800	1.9	12.4	1200.0	Photochemical, thermal
1000	1.6	9.2	1600.0	Thermal
1200	1.8	7.1	2200.0	Thermal

*In general, shorter wavelengths scatter and are absorbed more in tissues, limiting penetration depth. Data from Anderson and Parrish (1981) and Bashkatov et al. (2011)*.

Research on other parts of the electromagnetic spectrum is currently sparse but encouraging: *Enterococcus* (apparently resistant to blue light) has been shown to be sensitive to near- and mid-infrared (IR) light (Licata et al., 2015; D’Ercole et al., 2016), while the infectivity of *Chlamydiaceae* can be reduced with near-IR (Marti et al., 2015). Red light has been shown to reduce cell numbers in some pathogens (König et al., 2000; Martins et al., 2015; de Sousa et al., 2016), possibly due to the same porphyrin mechanism as blue light: porphyrins absorb most strongly in the blue region, but also absorb other visible wavelengths (Battisti et al., 2017). Additionally, infectivity of virus particles can be reduced by exposure to visible light (Richardson and Porter, 2005) Together, these data suggest that blue light is only one of a number of potential therapies, with the most obvious opportunities for development of antimicrobials exploiting other endogenous photosensitizers.

Flavins (Eichner et al., 2015; Makdoumi et al., 2017) and vitamin A (El-Agamey et al., 2017) can both be photosensitized to produce reactive oxygen. Other possibilities exist in the visible range, however – there are examples of visible and IR-photoinduced production of oxygen species in prokaryotes (Kohli and Gupta, 2003; Lubart et al., 2011) and eukaryotes (Hayashi et al., 1997; Karu, 2008), suggesting that suitable chromophores exist, although they have yet to be identified. DNA damage in red- and nIR-irradiated *Escherichia coli* has been shown to require other cellular components, rather than occurring as a direct effect of light interacting with DNA (Rocha Teixeira et al., 2014; Martins et al., 2015). While the chromophores in these cases are unknown, there is also molecular evidence for light-mediated cell damage. Ferritin is excited by visible light and can modify numerous substrates including proteins (Nikandrov et al., 1997; Saenz et al., 2016). As well as porphyrin, other tetrapyrroles may also have potential. Vitamin B12 is known to absorb in the visible region (Wang et al., 2015), while hematoporphyrin is used as a photosensitizer (Tanaka et al., 2011).

One noteworthy example of this principle is in the use of green light to treat the fungal infection onychomycosis. *Trichophyton rubrum*, one of the causative agents, produces a characteristic red pigment xanthomegnin (Gupta et al., 2000) which can be targeted with 532 nm light, causing significant inhibition of growth (Vural et al., 2008). Numerous wavelengths in the visible and infrared have been used to treat onychomycosis with some success (Gupta and Versteeg, 2017). As is the case with the use of blue light against bacteria, there remains little consensus on the mechanism of toxicity, or the optimal treatment wavelength, power, or duration.

Food spoilage organisms such as *Aspergillus* and *Phytophthora* are often pigmented, which may allow similar selective targeting. A significant body of work exists regarding the use of light as a sterilizing agent in food and water processing (Song et al., 2016; Fan et al., 2017), much of it based around ultraviolet light. UV, however, also penetrates poorly (**Table 1**), limiting its application to surface decolonization. Although UV predominates, visible light wavelengths have also been suggested for application in the food industry (Imada et al., 2014; Gunther et al., 2016). Thus, other antimicrobial wavelengths may find use in a range of applications.

## Oxygen-Independent Mechanisms

The reliance on oxygen intermediates for toxicity may also limit the application of blue light, with deep tissues and biofilms often microaerophilic or anaerobic. Many pathogens also possess sophisticated, protective oxidative stress responses, which can contribute to virulence (Coady et al., 2015; Cheng et al., 2017). In *Staphylococcus*, the presence of antioxidant carotenoid pigments such as staphyloxanthin affects the killing efficiency of blue light (Halstead et al., 2016). Similar carotenoids are induced by exposure to blue light in *Myxococcus* (Galbis-Martínez et al., 2012). Again, the applicability of antimicrobial light would be enhanced by identification of wavelengths with oxygen-independent toxicity.

Despite the well-known dangers of UV, more recent results suggest that the waveband should not be overlooked. UVC, which directly results in DNA damage, has been shown to be a very effective antimicrobial, reducing cell numbers with as little as 2 mJ cm^-2^ (Dean et al., 2011). Clinical applications have been trialed, with ultraviolet lighting shown to reduce surgical site infections (Ritter et al., 2007). The full range of ultraviolet wavelengths is little explored, with most studies employing broad-band sources. Certain specific wavelengths have been reported to offer selectivity for bacteria over mammalian cells (Buonanno et al., 2013; Narita et al., 2018), highlighting the need for studies with greater wavelength resolution. UV’s potent antimicrobial effect may be most easily applied to disinfection and sterilization, where patient compatibility is not required and positive results have already been seen (Anderson et al., 2017).

As well as DNA, bacterial proteins can be damaged irreversibly by UV light in a manner similar to that seen with oxidative damage (Bosshard et al., 2010). Other spectral bands may have similar effects, with exposure to green and red light altering protein folding, possibly by inducing reorganization of hydrogen bonds (Espinoza et al., 2015). Protein function can similarly be modulated by nIR light (Vojisavljevic et al., 2007), which in turn has been shown to cause DNA damage in plasmid DNA (Fonseca et al., 2012). Evidence for direct effects on cell components by wavelengths outside the ultraviolet range remains sparse, however, and the ubiquity of macromolecules such as DNA and protein may make selectivity against bacterial over host cells challenging (although potential targets exist). Metalloproteins (an emerging antibiotic target) may be potential targets here, being frequently virulence-associated and with characteristic absorbance properties (Dell’Acqua et al., 2011; Shumilina et al., 2014).

The killing mechanism of ultraviolet light is not entirely photochemical. Cells and spores have been shown to lyse under pulsed UV light as a result of localized transient temperature rise and water vaporization (Wekhof, 2000; Takeshita et al., 2003). The localized heating is dependent on higher absorption by the target cells than the surrounding environment (Fine and Gervais, 2004). In the case of UV light, DNA and amino acids are known to be absorptive chromophores, but other absorbers may also be identified. Successful development of this selective thermolysis approach depends on the identification of suitable bacterial chromophores and their activating wavelengths.

Biological macromolecules such as proteins (Barth, 2007), polysaccharides (Černá et al., 2003), and lipids (Hull et al., 2005) as well as small molecules (Amerov et al., 2004) all have characteristic absorbance spectra in the near- and mid-IR. Polysaccharides have broadly similar spectra but distinctive differences exist (Langkilde and Svantesson, 1995; Bekhit et al., 2016). Relatively minor chemical modifications can produce significant changes in absorbance (Hamcerencu et al., 2007), suggesting that the many variations found in bacterial capsules [*E. coli* alone has over 70 capsular subtypes (Whitfield, 2006)] may provide unique spectral differences to target. Indeed, bacteria (Tidwell et al., 2015; Almasoud et al., 2016) and fungi (Kogkaki et al., 2017) can be subtyped or differentiated from a eukaryotic host (Wang et al., 2010) by diagnostic fingerprint regions in their infrared spectra (Maity et al., 2013). Peptidoglycans, an obvious antimicrobial target, also have characteristic strain-specific spectra (Naumann et al., 1982). Such identifying peaks and regions, however, are by definition unique to particular species, suggesting that multiple therapeutic wavelengths could be required to maximize the range of possible target organisms.

Another approach to cellular disruption is the induction of damaging vibrational energy in the target. Such a disruption strategy may be of particular interest as an antiviral. The regular geometry of many viruses results in consistent vibrational frequencies (Dykeman and Sankey, 2010). If the intrinsic vibrational frequency of a viral particle is matched by that of an incident electromagnetic wave, the photons resonate and are absorbed, causing disruptive vibrations in the particle (Liu et al., 2009). This phenomenon can be exploited to destroy viruses with relatively low-powered microwave energy (Yang et al., 2015). It has been reported that very short (femtosecond) pulsed lasers can disrupt viruses and bacteria by a similar transfer of vibrational energy. The precise mechanisms underpinning this phenomenon are unclear, however, and findings to date have been inconsistent (Wigle et al., 2014) suggesting that considerable further work is required. Although not shown to reduce cell numbers, pulsed laser light has been shown to liberate biofilms from surfaces, possibly facilitating subsequent antibiotic therapy (Kizhner et al., 2011).

## Perspectives

Blue light undoubtedly has the potential to become a highly effective antimicrobial. Key questions remain to be answered, however, including around the mechanisms of toxicity and in particular the contribution of porphyrin-independent mechanisms. Opportunities are not limited to widely studied blue light, necessitating the continued exploration of other antimicrobial wavelengths. The development of alternative or complementary methods is vital to expanding the range of target organisms and clinical applications, as well as to reducing the risk of the development of resistance. To maximize efficacy, a realistic light-based therapy seems likely to require use of multiple wavelengths with several distinct targets.

While other possibilities for therapies certainly exist, their development is limited at present by a paucity of knowledge regarding properties such as absorbance, reflectance, and scatter in biological systems. The fundamental optical properties of bacterial cell components and of cells are all vital to exploiting the physicochemical (and resultant biological) interactions between light and cells but currently only understood in the context of a few specific systems. Even among those studies involving antimicrobial light, most are focused on a handful of narrow wavebands (Kim et al., 2013; Kumar et al., 2016). Thus, the vast majority of the electromagnetic spectrum remains to be explored but holds tremendous potential. The required research may be built on existing techniques and knowledge. Spectroscopic techniques are improving rapidly and come with a wealth of data about the absorptive and scattering properties of cells, which could be of great value.

Development of an optimal treatment regime also represents a significant barrier to translating research into the clinic. Current studies differ widely in their exploration of wavelength, power, and treatment duration, with very few giving evidence of an empirical process of optimization. Nevertheless, two complementary modalities prevail. High-power, short-duration treatments could be used for directed therapy such as wound disinfection, together with the use of lower powers to reduce resident bacterial load in wards or operating theaters. Either case brings unique challenges in terms of the relationship between lethality and required energy dose, much of which are still only poorly understood. Although widespread use of antimicrobial light may be limited by such practicalities or issues of dose and administration, even limited clinical implementation will assist in prolonging the lifespan of existing antibiotics. Moreover, as our understanding of the underlying mechanisms develop, opportunities for other applications such as in agriculture and food production are likely to present themselves and may lead to technological transformations in these industries. However, in order to maximize such opportunities, further research into the underlying science is a necessary requirement.

## Author Contributions

All authors listed have made a substantial, direct, and intellectual contribution to the work, and approved it for publication.

## Conflict of Interest Statement

The authors declare that the research was conducted in the absence of any commercial or financial relationships that could be construed as a potential conflict of interest.

## References

[B1] Almasoud N. Xu Y. Ellis D. I. Rooney P. Turton J. F. Goodacre R. (2016). Rapid discrimination of *Enterococcus faecium* strains using phenotypic analytical techniques. *Anal. Methods* 8 7603–7613. 10.1039/c6ay02326f

[B2] Amerov A. K. Chen J. Arnold M. A. (2004). Molar absorptivities of glucose and other biological molecules in aqueous solutions over the first overtone and combination regions of the near-infrared spectrum. *Appl. Spectrosc.* 58 1195–1204. 10.1366/0003702042336136 15527520

[B3] Anderson D. J. Chen L. F. Weber D. J. Moehring R. W. Lewis S. S. Triplett P. F. (2017). Enhanced terminal room disinfection and acquisition and infection caused by multidrug-resistant organisms and *Clostridium difficile* (the Benefits of Enhanced Terminal Room Disinfection study): a cluster-randomised, multicentre, crossover study. *Lancet* 389 805–814. 10.1016/S0140-6736(16)31588-4 28104287 PMC5935446

[B4] Anderson R. R. Parrish J. A. (1981). The optics of human skin. *J. Invest. Dermatol.* 77 13–19. 10.1111/1523-1747.ep12479191 7252245

[B5] Ashkenazi H. Malik Z. Harth Y. Nitzan Y. (2003). Eradication of *Propionibacterium acnes* by its endogenic porphyrins after illumination with high intensity blue light. *FEMS Immunol. Med. Microbiol.* 35 17–24. 10.1111/j.1574-695X.2003.tb00644.x 12589953

[B6] Barth A. (2007). Infrared spectroscopy of proteins. *Biochim. Biophys. Acta* 1767 1073–1101. 10.1016/j.bbabio.2007.06.004 17692815

[B7] Bashkatov A. N. Genina E. A. Tuchin V. V. (2011). Optical properties of skin, subcutaneous, and muscle tissues: a review. *J. Innov. Opt. Health Sci.* 04 9–38. 10.1142/S1793545811001319

[B8] Battisti A. Morici P. Ghetti F. Sgarbossa A. (2017). Spectroscopic characterization and fluorescence imaging of *Helicobacter pylori* endogenous porphyrins. *Biophys. Chem.* 229 19–24. 10.1016/j.bpc.2017.05.010 28576278

[B9] Bekhit M. Sánchez-González L. Ben Messaoud G. Desobry S. (2016). Design of microcapsules containing *Lactococcus lactis* subsp. *lactis* in alginate shell and xanthan gum with nutrients core. *LWT Food Sci. Technol.* 68 446–453. 10.1016/j.lwt.2015.12.037

[B10] Bertoloni G. Salvato B. Dall‘Acqua M. Vazzoler M. Jori G. (1984). Hematoporphyrin-sensitised photoinactivation of *Streptococcus faecalis*. *Photochem. Photobiol.* 39 811–816. 10.1111/j.1751-1097.1984.tb08864.x 6431458

[B11] Bhullar K. Waglechner N. Pawlowski A. Koteva K. Banks E. D. Johnston M. D. (2012). Antibiotic resistance is prevalent in an isolated cave microbiome. *PLOS ONE* 7:e34953. 10.1371/journal.pone.0034953 22509370 PMC3324550

[B12] Bosshard F. Riedel K. Schneider T. Geiser C. Bucheli M. Egli T. (2010). Protein oxidation and aggregation in UVA-irradiated *Escherichia coli* cells as signs of accelerated cellular senescence. *Environ. Microbiol.* 12 2931–2945. 10.1111/j.1462-2920.2010.02268.x 20545749

[B13] Bumah V. V. Aboualizadeh E. Masson-Meyers D. S. Eells J. T. Enwemeka C. S. Hirschmugl C. J. (2016). Spectrally resolved infrared microscopy and chemometric tools to reveal the interaction between blue light (470nm) and methicillin-resistant *Staphylococcus aureus*. *J. Photochem. Photobiol. B* 167 150–157. 10.1016/j.jphotobiol.2016.12.030 28064075

[B14] Bumah V. V. Whelan H. T. Masson-Meyers D. S. Quirk B. Buchmann E. Enwemeka C. S. (2015). The bactericidal effect of 470 nm light and hyperbaric oxygen on methicillin-resistant *Staphylococcus aureus* (MRSA). *Lasers Med. Sci.* 30 1153–1159. 10.1007/s10103-015-1722-9 25700768 PMC4535990

[B15] Buonanno M. Randers-Pehrson G. Bigelow A. W. Trivedi S. Lowy F. D. Spotnitz H. M. (2013). 207-nm UV light - a promising tool for safe low-cost reduction of surgical site infections. I: in vitro studies. *PLOS ONE* 8:e76968. 10.1371/journal.pone.0076968 24146947 PMC3797730

[B16] Černá M. Barros A. S. Nunes A. Rocha S. M. Delgadillo I. Čopíková J. (2003). Use of FT-IR spectroscopy as a tool for the analysis of polysaccharide food additives. *Carbohydr. Polym.* 51 383–389. 10.1016/S0144-8617(02)00259-X

[B17] Chang J. C. Ossoff S. F. Lobe D. C. Dorfman M. H. Dumais C. M. Qualls R. G. (1985). UV inactivation of pathogenic and indicator microorganisms. *Appl. Environ. Microbiol.* 49 1361–1365. 2990336 10.1128/aem.49.6.1361-1365.1985 PMC241729

[B18] Cheng C. Dong Z. Han X. Wang H. Jiang L. Sun J. (2017). Thioredoxin A is essential for motility and contributes to host infection of *Listeria monocytogenes* via redox interactions. *Front. Cell. Infect. Microbiol.* 7:287. 10.3389/fcimb.2017.00287 28702378 PMC5487381

[B19] Choi M. S. Yun S. J. Beom H. J. Park H. R. Lee J. B. (2011). Comparative Study of the bactericidal effects of 5-aminolevulinic acid with blue and red light on *Propionibacterium acnes*. *J. Dermatol.* 38 661–666. 10.1111/j.1346-8138.2010.01094.x 21352326

[B20] Cieplik F. Späth A. Leibl C. Gollmer A. Regensburger J. Tabenski L. (2014). Blue light kills *Aggregatibacter actinomycetemcomitans* due to its endogenous photosensitizers. *Clin. Oral Investig.* 18 1763–1769. 10.1007/s00784-013-1151-8 24297656

[B21] Coady A. Xu M. Phung Q. Cheung T. K. Bakalarski C. Alexander M. K. (2015). The *Staphylococcus aureus* ABC-type manganese transporter MntABC is critical for reinitiation of bacterial replication following exposure to phagocytic oxidative burst. *PLOS ONE* 10:e0138350. 10.1371/journal.pone.0138350 26379037 PMC4574778

[B22] Cobb C. M. (2006). Lasers in periodontics: a review of the literature. *J. Periodontol.* 77 545–564. 10.1902/jop.2006.050417 16584335

[B23] Dai T. Gupta A. Huang Y.-Y. Yin R. Murray C. K. Vrahas M. S. (2013). Blue light rescues mice from potentially fatal *Pseudomonas aeruginosa* burn infection: efficacy, safety, and mechanism of action. *Antimicrob. Agents Chemother.* 57 1238–1245. 10.1128/AAC.01652-12 23262998 PMC3591931

[B24] de Sousa D. L. Lima R. A. Zanin I. C. Klein M. I. Janal M. N. Duarte S. (2015). Effect of twice-daily blue light treatment on matrix-rich biofilm development. *PLOS ONE* 10:e0131941. 10.1371/journal.pone.0131941 26230333 PMC4521953

[B25] de Sousa N. T. Gomes R. C. Santos M. F. Brandino H. E. Martinez R. de Jesus Guirro R. R. (2016). Red and infrared laser therapy inhibits in vitro growth of major bacterial species that commonly colonize skin ulcers. *Lasers Med. Sci.* 31 549–556. 10.1007/s10103-016-1907-x 26886585

[B26] de Sousa N. T. Santos M. F. Gomes R. C. Brandino H. E. Martinez R. de Jesus Guirro R. R. (2015). Blue laser inhibits bacterial growth of *Staphylococcus aureus*, *Escherichia coli*, and *Pseudomonas aeruginosa*. *Photomed. Laser Surg.* 33 278–282. 10.1089/pho.2014.3854 25954830 PMC4432882

[B27] Dean S. J. Petty A. Swift S. McGhee J. J. Sharma A. Shah S. (2011). Efficacy and safety assessment of a novel ultraviolet C device for treating corneal bacterial infections. *Clin. Exp. Ophthalmol.* 39 156–163. 10.1111/j.1442-9071.2010.02471.x 21105972

[B28] Dell’Acqua S. Pauleta S. R. Moura I. Moura J. J. G. (2011). The tetranuclear copper active site of nitrous oxide reductase: the CuZ center. *J. Biol. Inorg. Chem.* 16 183–194. 10.1007/s00775-011-0753-3 21240533

[B29] D’Ercole S. Spoto G. Trentini P. Tripodi D. Petrini M. (2016). *In vitro* inactivation of *Enterococcus faecalis* with a LED Device. *J. Photochem. Photobiol. B* 160 172–177. 10.1016/j.jphotobiol.2016.04.015 27107704

[B30] Dykeman E. C. Sankey O. F. (2010). Atomistic modeling of the low-frequency mechanical modes and Raman spectra of icosahedral virus capsids. *Phys. Rev. E* 81:021918. 10.1103/PhysRevE.81.021918 20365606

[B31] Eichner A. Gollmer A. Späth A. Bäumler W. Regensburger J. König B. (2015). Fast and effective inactivation of *Bacillus atrophaeus* endospores using light-activated derivatives of vitamin B2. *Photochem. Photobiol. Sci.* 14 387–396. 10.1039/C4PP00285G 25423452

[B32] El-Agamey A. Melø T. B. Sliwka H.-R. (2017). Exploring the reactivity of retinol radical cation toward organic and biological molecules: a laser flash photolysis study. *J. Photochem. Photobiol. B* 170 33–39. 10.1016/j.jphotobiol.2017.03.009 28390257

[B33] Endarko E. Maclean M. Timoshkin I. V. MacGregor S. J. Anderson J. G. (2012). High-intensity 405 nm light inactivation of *Listeria monocytogenes*. *Photochem. Photobiol.* 88 1280–1286. 10.1111/j.1751-1097.2012.01173.x 22582879

[B34] Espinoza J. H. Reynaga-Hernández E. Ruiz-García J. Montero-Morán G. Sanchez-Dominguez M. Mercado-Uribe H. (2015). Effects of green and red light in βL-crystallin and ovalbumin. *Sci. Rep.* 5:18120. 10.1038/srep18120 26656181 PMC4677341

[B35] Fan X. Huang R. Chen H. (2017). Application of ultraviolet C technology for surface decontamination of fresh produce. *Trends Food Sci. Technol.* 70 9–19. 10.1016/j.tifs.2017.10.004

[B36] Feuerstein O. Ginsburg I. Dayan E. Veler D. Weiss E. I. (2005). Mechanism of visible light phototoxicity on *Porphyromonas gingivalis* and *Fusobacterium nucleatum*. *Photochem. Photobiol.* 81 1186–1189. 10.1562/2005-04-06-RA-477 15960594

[B37] Fine F. Gervais P. (2004). Efficiency of pulsed UV light for microbial decontamination of food powders. *J. Food Prot.* 67 787–792. 10.4315/0362-028X-67.4.787 15083732

[B38] Fonseca A. S. Geller M. Filho M. B. Valença S. S. de Paoli F. (2012). Low-level infrared laser effect on plasmid DNA. *Lasers Med. Sci.* 27 121–130. 10.1007/s10103-011-0905-2 21556926

[B39] Galbis-Martínez M. Padmanabhan S. Murillo F. J. Elías-Arnanz M. (2012). CarF mediates signaling by singlet oxygen, generated via photoexcited protoporphyrin IX, in *Myxococcus xanthus* light-induced carotenogenesis. *J. Bacteriol.* 194 1427–1436. 10.1128/JB.06662-11 22267513 PMC3294824

[B40] Goff D. A. Kullar R. Goldstein E. J. C. Gilchrist M. Nathwani D. Cheng A. C. (2017). A global call from five countries to collaborate in antibiotic stewardship: united we succeed, divided we might fail. *Lancet Infect. Dis.* 17 e56–e63. 10.1016/S1473-3099(16)30386-3 27866945

[B41] Gourmelon M. Cillard J. Pommepuy M. (1994). Visible light damage to *Escherichia coli* in seawater: oxidative stress hypothesis. *J. Appl. Bacteriol.* 77 105–112. 10.1111/j.1365-2672.1994.tb03051.x 7928776

[B42] Guffey J. S. Payne W. Jones T. Martin K. (2013). Evidence of resistance development by *Staphylococcus aureus* to an in vitro, multiple stage application of 405 nm light from a supraluminous diode array. *Photomed. Laser Surg.* 31 179–182. 10.1089/pho.2012.3450 23484587

[B43] Gunther N. W. Phillips J. G. Sommers C. (2016). The effects of 405-nm visible light on the survival of *Campylobacter* on chicken skin and stainless steel. *Foodborne Pathog. Dis.* 13 245–250. 10.1089/fpd.2015.2084 26938455

[B44] Gupta A. K. Ahmad I. Borst I. Summerbell R. C. (2000). Detection of xanthomegnin in epidermal materials infected with *Trichophyton rubrum*. *J. Invest. Dermatol.* 115 901–905. 10.1046/j.1523-1747.2000.00150.x 11069630

[B45] Gupta A. K. Versteeg S. G. (2017). A critical review of improvement rates for laser therapy used to treat toenail onychomycosis. *J. Eur. Acad. Dermatol. Venereol.* 31 1111–1118. 10.1111/jdv.14212 28294418

[B46] Gupta S. Maclean M. Anderson J. G. MacGregor S. J. Meek R. M. D. Grant M. H. (2015). Inactivation of micro-organisms isolated from infected lower limb arthroplasties using high-intensity narrow-spectrum (HINS) light. *Bone Joint J.* 97-B, 283–288. 10.1302/0301-620X.97B2.35154 25628296

[B47] Halstead F. D. Thwaite J. E. Burt R. Laws T. R. Raguse M. Moeller R. (2016). Antibacterial activity of blue light against nosocomial wound pathogens growing planktonically and as mature biofilms. *Appl. Environ. Microbiol.* 82 4006–4016. 10.1128/AEM.00756-16 27129967 PMC4907187

[B48] Hamblin M. R. Viveiros J. Yang C. Ahmadi A. Ganz R. A. Tolkoff M. J. (2005). *Helicobacter pylori* accumulates photoactive porphyrins and is killed by visible light. *Antimicrob. Agents Chemother.* 49 2822–2827. 10.1128/AAC.49.7.2822-2827.2005 15980355 PMC1168670

[B49] Hamcerencu M. Desbrieres J. Popa M. Khoukh A. Riess G. (2007). New unsaturated derivatives of xanthan gum: synthesis and characterization. *Polymer* 48 1921–1929. 10.1016/j.polymer.2007.01.048

[B50] Hayashi S. Ishimoto S. Wu G. Wee W. Rao N. McDonnell P. (1997). Oxygen free radical damage in the cornea after excimer laser therapy. *Br. J. Ophthalmol.* 81 141–144. 10.1136/bjo.81.2.141 9059249 PMC1722119

[B51] Henry C. A. Judy M. Dyer B. Wagner M. Matthews J. L. (1995). Sensitivity of *Porphyromonas* and *Prevotella* species in liquid media to argon laser. *Photochem. Photobiol.* 61 410–413. 10.1111/j.1751-1097.1995.tb08631.x 7740086

[B52] Hill R. Healy B. Holloway L. Kuncic Z. Thwaites D. Baldock C. (2014). Advances in kilovoltage x-ray beam dosimetry. *Phys. Med. Biol.* 59:R183. 10.1088/0031-9155/59/6/R183 24584183

[B53] Hiraku Y. Ito K. Hirakawa K. Kawanishi S. (2007). Photosensitized DNA damage and its protection via a novel mechanism. *Photochem. Photobiol.* 83 205–212. 10.1562/2006-03-09-IR-840 16965181

[B54] Hull M. C. Cambrea L. R. Hovis J. S. (2005). Infrared spectroscopy of fluid lipid bilayers. *Anal. Chem.* 77 6096–6099. 10.1021/ac050990c 16159147

[B55] Imada K. Tanaka S. Ibaraki Y. Yoshimura K. Ito S. (2014). Antifungal effect of 405-nm light on *Botrytis cinerea*. *Lett. Appl. Microbiol.* 59 670–676. 10.1111/lam.12330 25236427

[B56] Jawetz E. (1963). Antibiotics revisited: problems and prospects after two decades. *Br. Med. J.* 2 951–955. 10.1136/bmj.2.5363.951 14056921 PMC1873080

[B57] Jin J.-Y. Lee S.-H. Yoon H.-J. (2010). A comparative study of wound healing following incision with a scalpel, diode laser or Er,Cr:YSGG laser in guinea pig oral mucosa: a histological and immunohistochemical analysis. *Acta Odontol. Scand.* 68 232–238. 10.3109/00016357.2010.492356 20513169

[B58] Karu T. I. (2008). Mitochondrial signaling in mammalian cells activated by red and near-IR radiation. *Photochem. Photobiol.* 84 1091–1099. 10.1111/j.1751-1097.2008.00394.x 18651871

[B59] Kim M.-J. Yuk H.-G. (2017). Antibacterial mechanism of 405-nanometer light-emitting diode against *Salmonella* at refrigeration temperature. *Appl. Environ. Microbiol.* 83:e02582-16. 10.1128/AEM.02582-16 28003197 PMC5311417

[B60] Kim S. Kim J. Lim W. Jeon S. Kim O. Koh J.-T. (2013). *In vitro* bactericidal effects of 625 525 and 425 nm wavelength (Red, Green, and Blue) light-emitting diode irradiation. *Photomed. Laser Surg.* 31 554–562. 10.1089/pho.2012.3343 24138193 PMC3818000

[B61] Kizhner V. Krespi Y. P. Hall-Stoodley L. Stoodley P. (2011). Laser-generated shockwave for clearing medical device biofilms. *Photomed. Laser Surg.* 29 277–282. 10.1089/pho.2010.2788 21182450

[B62] Kjeldstad B. (1987). Different photoinactivation mechanisms in *Propionibacterium acnes* for near-ultraviolet and visible light. *Photochem. Photobiol.* 46 363–366. 10.1111/j.1751-1097.1987.tb04782.x 3671513

[B63] Kjeldstad B. Johnsson A. (1986). An action spectrum for blue and near ultraviolet in activation of *Propionibacterium acnes*; with emphasis on a possible porphyrin photosensitization. *Photochem. Photobiol.* 43 67–70. 10.1111/j.1751-1097.1986.tb05592.x 3952162

[B64] Koenig K. Rueck A. C. Schneckenburger H. (1992). Fluorescence detection and photodynamic activity of endogenous protoporphyrin in human skin. *Opt. Eng.* 31 1470–1475. 10.1117/12.57700

[B65] Kogkaki E. A. Sofoulis M. Natskoulis P. Tarantilis P. A. Pappas C. S. Panagou E. Z. (2017). Differentiation and identification of grape-associated black aspergilli using Fourier transform infrared (FT-IR) spectroscopic analysis of mycelia. *Int. J. Food Microbiol.* 259 22–28. 10.1016/j.ijfoodmicro.2017.07.020 28779624

[B66] Kohli R. Gupta P. K. (2003). Irradiance dependence of the He–Ne laser-induced protection against UVC radiation in *E. coli* strains. *J. Photochem. Photobiol. B* 69 161–167. 10.1016/S1011-1344(03)00018-6 12695030

[B67] König K. Teschke M. Sigusch B. Glockmann E. Eick S. Pfister W. (2000). Red light kills bacteria via photodynamic action. *Cell. Mol. Biol.* 46 1297–1303. 11075959

[B68] Kumar A. Ghate V. Kim M. J. Zhou W. Khoo G. H. Yuk H. G. (2016). Antibacterial efficacy of 405, 460 and 520 nm light emitting diodes on *Lactobacillus plantarum*, *Staphylococcus aureus* and *Vibrio parahaemolyticus*. *J. Appl. Microbiol.* 120 49–56. 10.1111/jam.12975 26481103

[B69] Kumar S. R. Imlay J. A. (2013). How *Escherichia coli* tolerates profuse hydrogen peroxide formation by a catabolic pathway. *J. Bacteriol.* 195 4569–4579. 10.1128/JB.00737-13 23913322 PMC3807432

[B70] Langkilde F. W. Svantesson A. (1995). Identification of celluloses with Fourier-transform (FT) mid-infrared, FT-Raman and near-infrared spectrometry. *J. Pharm. Biomed. Anal.* 13 409–414. 10.1016/0731-7085(95)01298-Y 9696549

[B71] Lembo A. J. Ganz R. A. Sheth S. Cave D. Kelly C. Levin P. (2009). Treatment of *Helicobacter pylori* infection with intra-gastric violet light phototherapy – a pilot clinical trial. *Lasers Surg. Med.* 41 337–344. 10.1002/lsm.20770 19533762 PMC2841969

[B72] Licata M. E. Albanese A. Campisi G. Geraci D. M. Russo R. Gallina G. (2015). Effectiveness of a new method of disinfecting the root canal, using Er, Cr:YSGG laser to kill *Enterococcus faecalis* in an infected tooth model. *Lasers Med. Sci.* 30 707–712. 10.1007/s10103-013-1410-6 23917414

[B73] Ling L. L. Schneider T. Peoples A. J. Spoering A. L. Engels I. Conlon B. P. (2015). A new antibiotic kills pathogens without detectable resistance. *Nature* 517 455–459. 10.1038/nature14098 25561178 PMC7414797

[B74] Liu T. M. Chen H. P. Wang L. T. Wang J. R. Luo T. N. Chen Y. J. (2009). Microwave resonant absorption of viruses through dipolar coupling with confined acoustic vibrations. *Appl. Phys. Lett.* 94:043902. 10.1063/1.3074371 28676694

[B75] Lubart R. Lipovski A. Nitzan Y. Friedmann H. (2011). A possible mechanism for the bactericidal effect of visible light. *Laser Ther.* 20 17–22. 10.5978/islsm.20.17 24155508 PMC3806074

[B76] Lyon B. R. Skurray R. (1987). Antimicrobial resistance of *Staphylococcus aureus*: genetic basis. *Microbiol. Rev.* 51 88–134. 3031442 10.1128/mr.51.1.88-134.1987 PMC373094

[B77] Maclean M. Booth M. Anderson J. MacGregor S. Woolsey G. Coia J. (2013). Continuous decontamination of an intensive care isolation room during patient occupancy using 405 nm light technology. *J. Infect. Prev.* 14 176–181. 10.1177/1757177413483646

[B78] Maclean M. MacGregor S. J. Anderson J. G. Woolsey G. (2008a). High-intensity narrow-spectrum light inactivation and wavelength sensitivity of *Staphylococcus aureus*. *FEMS Microbiol. Lett.* 285 227–232. 10.1111/j.1574-6968.2008.01233.x 18557942

[B79] Maclean M. MacGregor S. J. Anderson J. G. Woolsey G. A. (2008b). The role of oxygen in the visible-light inactivation of *Staphylococcus aureus*. *J. Photochem. Photobiol. B* 92 180–184. 10.1016/j.jphotobiol.2008.06.006 18657991

[B80] Maclean M. MacGregor S. J. Anderson J. G. Woolsey G. (2009). Inactivation of bacterial pathogens following exposure to light from a 405-nanometer light-emitting diode array. *Appl. Environ. Microbiol.* 75 1932–1937. 10.1128/AEM.01892-08 19201962 PMC2663198

[B81] Maity J. P. Kar S. Lin C.-M. Chen C.-Y. Chang Y.-F. Jean J.-S. (2013). Identification and discrimination of bacteria using Fourier transform infrared spectroscopy. *Spectrochim. Acta. A Mol. Biomol. Spectrosc.* 116 478–484. 10.1016/j.saa.2013.07.062 23973597

[B82] Makdoumi K. Goodrich R. Bäckman A. (2017). Photochemical eradication of methicillin-resistant *Staphylococcus aureus* by blue light activation of riboflavin. *Acta Ophthalmol.* 95 498–502. 10.1111/aos.13409 28205348

[B83] Manaia C. M. (2017). Assessing the risk of antibiotic resistance transmission from the environment to humans: non-direct proportionality between abundance, and risk. *Trends Microbiol.* 25 173–181. 10.1016/j.tim.2016.11.014 28012687

[B84] Marti H. Blenn C. Borel N. (2015). The contribution of temperature, exposure intensity and visible light to the inhibitory effect of irradiation on acute chlamydial infection. *J. Photochem. Photobiol. B* 153 324–333. 10.1016/j.jphotobiol.2015.10.012 26513384

[B85] Martins W. A. Polignano G. A. C. Guimarães O. R. Geller M. Paoli F. Fonseca A. S. (2015). Dichromatic laser radiation effects on DNA of *Escherichia coli* and plasmids. *Laser Phys.* 25:045603 10.1088/1054-660X/25/4/045603

[B86] Møller K. I. Kongshoj B. Philipsen P. A. Thomsen V. O. Wulf H. C. (2005). How Finsen’s light cured lupus vulgaris. *Photodermatol. Photoimmunol. Photomed.* 21 118–124. 10.1111/j.1600-0781.2005.00159.x 15888127

[B87] Mussi M. A. Gaddy J. A. Cabruja M. Arivett B. A. Viale A. M. Rasia R. (2010). The opportunistic human pathogen *Acinetobacter baumannii* senses and responds to light. *J. Bacteriol.* 192 6336–6345. 10.1128/JB.00917-10 20889755 PMC3008525

[B88] Narita K. Asano K. Morimoto Y. Igarashi T. Hamblin M. R. Dai T. (2018). Disinfection and healing effects of 222-nm UVC light on methicillin-resistant *Staphylococcus aureus* infection in mouse wounds. *J. Photochem. Photobiol. B* 178 10–18. 10.1016/j.jphotobiol.2017.10.030 29101868 PMC5771808

[B89] Naumann D. Barnickel G. Bradaczek H. Labischinski H. Giesbrecht P. (1982). Infrared spectroscopy, a tool for probing bacterial peptidoglycan. *Eur. J. Biochem.* 125 505–515. 10.1111/j.1432-1033.1982.tb06711.x 7117249

[B90] Neu H. C. (1992). The crisis in antibiotic resistance. *Science* 257 1064–1073. 10.1126/science.257.5073.1064 1509257

[B91] Nikandrov V. V. Grätzel C. K. Moser J.-E. Grätzel M. (1997). Light induced redox reactions involving mammalian ferritin as photocatalyst. *J. Photochem. Photobiol. B* 41 83–89. 10.1016/S1011-1344(97)00085-7 9440316

[B92] Nitzan Y. Salmon-Divon M. Shporen E. Malik Z. (2004). ALA induced photodynamic effects on Gram positive and negative bacteria. *Photochem. Photobiol. Sci.* 3 430–435. 10.1039/B315633H 15122360

[B93] Nitzan Y. Shainberg B. Malik Z. (1987). Photodynamic effects of deuteroporphyrin on Gram-positive bacteria. *Curr. Microbiol.* 15 251–258. 10.1007/BF01589376

[B94] Ramakrishnan P. Maclean M. MacGregor S. J. Anderson J. G. Grant M. H. (2014). Differential sensitivity of osteoblasts and bacterial pathogens to 405-nm light highlighting potential for decontamination applications in orthopedic surgery. *J. Biomed. Opt.* 19 105001–105001. 10.1117/1.JBO.19.10.105001 25277146

[B95] Ramakrishnan P. Maclean M. MacGregor S. J. Anderson J. G. Grant M. H. (2016). Cytotoxic responses to 405 nm light exposure in mammalian and bacterial cells: involvement of reactive oxygen species. *Toxicol. In Vitro* 33 54–62. 10.1016/j.tiv.2016.02.011 26916085

[B96] Richardson T. B. Porter C. D. (2005). Inactivation of murine leukaemia virus by exposure to visible light. *Virology* 341 321–329. 10.1016/j.virol.2005.07.025 16099012

[B97] Ritter M. A. Olberding E. M. Malinzak R. A. (2007). Ultraviolet lighting during orthopaedic surgery and the rate of infection. *J. Bone Joint Surg. Am.* 89 1935–1940. 10.2106/JBJS.F.01037 17768189

[B98] Rocha Teixeira G. da Silva Marciano R. da Silva Sergio L. P. Castanheira Polignano G. A. Roberto Guimarães O. Geller M. (2014). Infrared laser effects at fluences used for treatment of dentin hypersensitivity on DNA repair in *Escherichia coli* and plasmids. *Opt. Laser Technol.* 64 46–52. 10.1016/j.optlastec.2014.04.023

[B99] Russell A. (2003). Biocide use and antibiotic resistance: the relevance of laboratory findings to clinical and environmental situations. *Lancet Infect. Dis.* 3 794–803. 10.1016/S1473-3099(03)00833-8 14652205

[B100] Saenz N. Sánchez M. Gálvez N. Carmona F. Arosio P. Dominguez-Vera J. M. (2016). Insights on the (auto)photocatalysis of ferritin. *Inorg. Chem.* 55 6047–6050. 10.1021/acs.inorgchem.6b00547 27265598

[B101] Sailer R. Strauss W. S. L. König K. Rück A. Steiner R. (1997). Correlation between porphyrin biosynthesis and photodynamic inactivation of *Pseudomonas aeruginosa* after incubation with 5-aminolaevulinic amid. *J. Photochem. Photobiol. B* 39 236–242. 10.1016/S1011-1344(96)00019-X

[B102] Schroeder M. Brooks B. D. Brooks A. E. (2017). The complex relationship between virulence and antibiotic resistance. *Genes* 8:39. 10.3390/genes8010039 28106797 PMC5295033

[B103] Shumilina E. Dobrovolska O. Conte R. D. Holen H. W. Dikiy A. (2014). Competitive cobalt for zinc substitution in mammalian methionine sulfoxide reductase B1 overexpressed in *E. coli*: structural and functional insight. *J. Biol. Inorg. Chem.* 19 85–95. 10.1007/s00775-013-1064-7 24271273 PMC3889830

[B104] Song K. Mohseni M. Taghipour F. (2016). Application of ultraviolet light-emitting diodes (UV-LEDs) for water disinfection: a review. *Water Res.* 94 341–349. 10.1016/j.watres.2016.03.003 26971809

[B105] Takeshita K. Shibato J. Sameshima T. Fukunaga S. Isobe S. Arihara K. (2003). Damage of yeast cells induced by pulsed light irradiation. *Int. J. Food Microbiol.* 85 151–158. 10.1016/S0168-1605(02)00509-3 12810279

[B106] Tanaka M. Kinoshita M. Yoshihara Y. Shinomiya N. Seki S. Nemoto K. (2011). Photodynamic therapy using intra-articular photofrin for murine MRSA arthritis: biphasic light dose response for neutrophil-mediated antibacterial effect. *Lasers Surg. Med.* 43 221–229. 10.1002/lsm.21037 21412806 PMC3071702

[B107] Tanzi E. L. Lupton J. R. Alster T. S. (2003). Lasers in dermatology: four decades of progress. *J. Am. Acad. Dermatol.* 49 1–34. 10.1067/mjd.2003.582 12833005

[B108] Tidwell J. E. Dawson-Andoh B. Adedipe E. O. Nkansah K. Dietz M. J. (2015). Can near-infrared spectroscopy detect and differentiate implant-associated biofilms? *Clin. Orthop. Relat. Res.* 473 3638–3646. 10.1007/s11999-015-4497-1 26265208 PMC4586235

[B109] Tomb R. M. Maclean M. Coia J. E. MacGregor S. J. Anderson J. G. (2017). Assessment of the potential for resistance to antimicrobial violet-blue light in *Staphylococcus aureus*. *Antimicrob. Resist. Infect. Control* 6:100. 10.1186/s13756-017-0261-5 29046782 PMC5639585

[B110] Tschowri N. Busse S. Hengge R. (2009). The BLUF-EAL protein YcgF acts as a direct anti-repressor in a blue-light response of *Escherichia coli*. *Genes Dev.* 23 522–534. 10.1101/gad.499409 19240136 PMC2648647

[B111] Tsiodras S. Gold H. S. Sakoulas G. Eliopoulos G. M. Wennersten C. Venkataraman L. (2001). Linezolid resistance in a clinical isolate of *Staphylococcus aureus*. *Lancet* 358 207–208. 10.1016/S0140-6736(01)05410-1 11476839

[B112] van der Meulen F. W. Ibrahim K. Sterenborg H. J. Alphen L. V. Maikoe A. Dankert J. (1997). Photodynamic destruction of *Haemophilus parainfluenzae* by endogenously produced porphyrins. *J. Photochem. Photobiol. B* 40 204–208. 10.1016/S1011-1344(97)00057-2 9372610

[B113] Vila J. Ruiz J. Marco F. Barcelo A. Goñi P. Giralt E. (1994). Association between double mutation in gyrA gene of ciprofloxacin-resistant clinical isolates of *Escherichia coli* and MICs. *Antimicrob. Agents Chemother.* 38 2477–2479. 10.1128/AAC.38.10.2477 7840592 PMC284767

[B114] Vojisavljevic V. Pirogova E. Cosic I. (2007). The effect of electromagnetic radiation (550 – 850 nm) on l-lactate dehydrogenase kinetics. *Int. J. Radiat. Biol.* 83 221–230. 10.1080/09553000701227565 17575949

[B115] von Wintersdorff C. J. Penders J. van Niekerk J. M. Mills N. D. Majumder S. van Alphen L. B. (2016). Dissemination of antimicrobial resistance in microbial ecosystems through horizontal gene transfer. *Front. Microbiol.* 7:173 10.3389/fmicb.2016.00173 PMC4759269 26925045

[B116] Vural E. Winfield H. L. Shingleton A. W. Horn T. D. Shafirstein G. (2008). The effects of laser irradiation on *Trichophyton rubrum* growth. *Lasers Med. Sci.* 23 349–353. 10.1007/s10103-007-0492-4 17902014

[B117] Wainwright M. Maisch T. Nonell S. Plaetzer K. Almeida A. Tegos G. P. (2016). Photoantimicrobials—are we afraid of the light? *Lancet Infect. Dis.* 17 e49–e55. 10.1016/S1473-3099(16)30268-7 27884621 PMC5280084

[B118] Wang J. Kim K. H. Kim S. Kim Y. S. Li Q. X. Jun S. (2010). Simple quantitative analysis of *Escherichia coli* K-12 internalized in baby spinach using Fourier transform infrared spectroscopy. *Int. J. Food Microbiol.* 144 147–151. 10.1016/j.ijfoodmicro.2010.09.013 20937537

[B119] Wang J. Wei J. Su S. Qiu J. (2015). Novel fluorescence resonance energy transfer optical sensors for vitamin B 12 detection using thermally reduced carbon dots. *New J. Chem.* 39 501–507. 10.1039/C4NJ00538D

[B120] Wang Y. Wu X. Chen J. Amin R. Lu M. Bhayana B. (2016). Antimicrobial blue light inactivation of Gram-negative pathogens in biofilms: in vitro and in vivo studies. *J. Infect. Dis.* 213 1380–1387. 10.1093/infdis/jiw070 26908743 PMC4813746

[B121] Wekhof A. (2000). Disinfection with flash lamps. *PDA J. Pharm. Sci. Technol.* 54 264–276. 10927918

[B122] Whitfield C. (2006). Biosynthesis and assembly of capsular polysaccharides in *Escherichia coli*. *Annu. Rev. Biochem.* 75 39–68. 10.1146/annurev.biochem.75.103004.142545 16756484

[B123] Wigle J. C. Holwitt E. A. Estlack L. E. Noojin G. D. Saunders K. E. Yakovlev V. V. (2014). No effect of femtosecond laser pulses on M13, *E. coli*, DNA, or protein. *J. Biomed. Opt.* 19:015008. 10.1117/1.JBO.19.1.015008 24474502

[B124] Yang P. Wang N. Wang C. Yao Y. Fu X. Yu W. (2017). 460 nm visible light irradiation eradicates MRSA via inducing prophage activation. *J. Photochem. Photobiol. B* 166 311–322. 10.1016/j.jphotobiol.2016.12.001 28024282

[B125] Yang S.-C. Lin H.-C. Liu T.-M. Lu J.-T. Hung W.-T. Huang Y.-R. (2015). Efficient structure resonance energy transfer from microwaves to confined acoustic vibrations in viruses. *Sci. Rep.* 5:18030. 10.1038/srep18030 26647655 PMC4673452

[B126] Zhu H. Kochevar I. E. Behlau I. Zhao J. Wang F. Wang Y. (2017). Antimicrobial blue light therapy for infectious keratitis: ex vivo and in vivo studies. *Invest. Ophthalmol. Vis. Sci.* 58 586–593. 10.1167/iovs.16-20272 28129422 PMC5283079

[B127] Zipperer A. Konnerth M. C. Laux C. Berscheid A. Janek D. Weidenmaier C. (2016). Human commensals producing a novel antibiotic impair pathogen colonization. *Nature* 535 511–516. 10.1038/nature18634 27466123

